# Digital Competencies for Pediatric Nurse Leaders to Sustain Patient- and Family-Centered Care: An Interpretative Phenomenological Analysis

**DOI:** 10.3390/healthcare14101303

**Published:** 2026-05-11

**Authors:** Alaa Hussain Hafiz

**Affiliations:** Department of Maternity and Child Health Nursing, Faculty of Nursing, King Abdulaziz University, Jeddah 24123, Saudi Arabia; ahhafidh@kau.edu.sa

**Keywords:** pediatric nursing, nursing leadership, digital health competency, patient-centered care, family-centered care, electronic health records, telehealth, culturally responsive care, psychological safety, qualitative research

## Abstract

**Background/Objectives:** Digital systems are being increasingly used to mediate pediatric care, yet many competency models remain predominantly technical and may unintentionally dilute patient- and family-centered care. This study aimed to identify empirically grounded digital competencies that enable pediatric nurse leaders to sustain patient- and family-centered care and to propose a practice-ready competency map. **Methods:** An interpretative phenomenological study was conducted across three hospitals in Saudi Arabia, purposively selected for varying levels of digital maturity. Ten pediatric nurse leaders completed two in-depth, semi-structured interviews (60–90 min) and a four-week reflective journal. Data were analyzed ideographically and then across cases using interpretative phenomenological analysis. Interviews were conducted in Arabic or English; translation included professional translation, partial back-translation (30%), and bilingual review. **Results:** Four interlinked competency domains emerged: (1) Relational digital presence, co-viewing the electronic health record, narrating documentation, and coordinating gaze and screen use to preserve relational connection; (2) Vulnerable expertise, micro-coaching at the point of care and transparent discussion of near-misses to build psychological safety; (3) Culturally legible communication, multimodal, language-congruent communication and explicit boundaries for sensitive information; and (4) Judgment-with-data, documenting override rationales and balancing algorithmic indicators with contextual family need. Together, these domains formed a screen-side competency map translating lived experience into trainable micro-practices. **Conclusions:** Digital competence in pediatric nursing leadership is relational, culturally situated, and clinically interpretive rather than a linear technical checklist. Embedding these competencies into leadership development and digital workflow design may help protect and strengthen patient- and family-centered care in technology-mediated pediatric care.

## 1. Introduction

Digital technologies are now embedded in routine healthcare delivery, reshaping how nurses document, communicate, coordinate care, and support clinical decisions [1]. This shift is particularly consequential in pediatrics, where care is inherently relational and frequently unfolds through triadic encounters among clinicians, children, and families [2,3]. Electronic health records (EHRs), telehealth platforms, patient portals, and emerging AI-enabled tools can enhance access, continuity, and data-informed decision-making; however, they may also reconfigure attention, conversational flow, and the conditions under which families participate meaningfully in care [4]. In practice, the clinical encounter is increasingly mediated by screens, templates, and algorithmic prompts, quiet forces that can shape what is noticed, what is documented, and what is left unsaid [5,6].

Patient- and Family-Centered Care (PFCC) is a foundational philosophy in pediatric nursing that positions families as essential partners in a child’s health and well-being [7,8]. PFCC is commonly operationalized through four interrelated principles: dignity and respect, information sharing, participation, and collaboration [9]. In pediatric settings where children may not be able to fully articulate needs or preferences, PFCC is central to safe, developmentally appropriate care because it integrates families’ knowledge of the child with clinical expertise [10]. Evidence links PFCC-oriented care to improved experiences of care and higher satisfaction among children and families, reinforcing its centrality to pediatric practice [11]. Professional pediatric bodies further emphasize that high-quality pediatric care cannot be delivered in isolation from the family system, consolidating PFCC as a defining standard for pediatric services [12]. Accordingly, digital innovation in pediatrics must be evaluated not only for functionality and efficiency, but also for its effects on family partnership, communication, and respectful engagement [7,13].

Despite growing recognition of the need for digitally competent nurse leadership, current competency frameworks offer limited pediatric-specific or relational guidance. The European DigComp 2.2 framework provides broad digital literacy descriptors, but is not designed for clinical leadership or relational care contexts [14]. The TIGER (Technology Informatics Guiding Education Reform) nursing informatics competency model addresses informatics knowledge and skills but devotes limited attention to relational presence, cultural situatedness, or pediatric-specific demands [15,16]. The American Organization for Nursing Leadership (AONL) Nurse Leader Digital Transformation guidelines foreground strategic and governance competencies but offer less specificity regarding how leaders should enact digitally competent practice in family-centered bedside encounters [17]. Each of these frameworks thus leaves a gap: the relational, developmental, and culturally situated dimensions of leadership practice required to sustain patient- and family-centered care (PFCC) in pediatric digital environments remain underspecified [18]. In this paper, digital competency is conceptualized as enacted, situated professional practice, with the capacity to perform digital work in ways that are relationally, culturally, and clinically appropriate rather than as a fixed skills inventory.

In the Saudi Arabian tertiary pediatric care context in which this study was conducted, PFCC is operationalized within a healthcare system undergoing rapid digital transformation under Vision 2030 eHealth priorities, including national electronic health record rollout and telehealth expansion [12,19]. Culturally, PFCC in this setting is reflected by extended family involvement in clinical decision-making [20], Arabic as the primary care language, established norms around interpersonal over digitally mediated disclosure, and gender dynamics that shape communication in clinical encounters [18,21]. These features mean that the four internationally recognized PFCC principles, dignity and respect, information sharing, participation, and collaboration, must be enacted through culturally specific communicative practices, a layer of complexity that is only partly addressed by generic digital competency frameworks [22,23,24]. Understanding how pediatric nurse leaders navigate this complexity is therefore both theoretically important and practically urgent.

Digital health technologies increasingly structure pediatric care delivery, including EHRs, telehealth systems, mobile applications, and AI-enabled decision support [25]. For children with chronic conditions, connected monitoring can enable earlier detection of deterioration and more proactive support [25]. Other modalities, including immersive technologies for symptom management and biosensors for continuous monitoring, extend the scope of interventions and the contexts in which monitoring occurs [26]. Collectively, these tools may enhance coordination and continuity while expanding families’ opportunities to access information and participate across settings [27]. Yet digitization also introduces interactional and equity risks that are highly salient in pediatrics [28]. During encounters, clinicians’ attention to documentation and system navigation can reduce eye contact and interrupt turn-taking [29], and constrain meaningful conversation with children and families [30]. Technology-mediated practice may also widen disparities when families face barriers related to digital access, literacy, language, or comfort with online communication [31,32]. This creates an enduring implementation tension: how to realize the benefits of digital innovation while sustaining the empathic, developmentally attuned communication and partnership that PFCC requires [33].

How this tension is navigated depends heavily on pediatric nurse leadership [34]. Nurse leaders influence how technologies are selected, implemented, and routinized within workflows, and how they are evaluated; they also shape the conditions under which staff enact PFCC in technology-mediated practice [35]. Leadership guidance emphasizes competencies such as advocacy, communication, collaboration, and systems thinking, which must increasingly be enacted through digital systems and digitally mediated channels [36]. Nurse-led digital transformation underscores nurses’ strategic role in implementation and optimization, and their proximity to children and families positions them to advocate for PFCC-aligned workflows and patient- and family-centered technology design [37,38]. However, effective leadership in this space requires competencies beyond traditional leadership skills, including digital literacy, technology-enabled change management, and the practical translation of PFCC principles into expectations for documentation, communication, and decision support in everyday care [39].

Despite broad recognition of the need for digitally competent nursing leadership, there remains limited empirical clarity regarding which digital competencies pediatric nurse leaders draw upon to sustain PFCC and how these competencies are enacted in technology-mediated clinical environments [40]. Available digital competency frameworks are often generic and may insufficiently account for the relational and developmental complexities of pediatrics or specify the practices required to preserve PFCC when care is mediated by screens, portals, and decision-support prompts [41,42]. Even widely used competency approaches offer limited pediatric-specific guidance on maintaining family partnerships and culturally responsive communication while navigating complex digital systems [40]. Furthermore, qualitative evidence remains limited regarding how pediatric nurse leaders make sense of digital leadership, how they navigate relational and ethical tensions during digitization, and which competencies they rely on to uphold PFCC in practice [43,44,45]. Addressing this gap is essential to inform leadership development, implementation strategies, and system design that protect PFCC as pediatric care becomes increasingly technology-mediated.

### 1.1. Aim of the Study

This study aims to explore how pediatric nurse leaders make sense of digital leadership in technology-mediated practice and to identify the digital competencies they describe as essential for sustaining Patient- and Family-Centered Care (PFCC) across pediatric care settings.

### 1.2. Research Question

How do pediatric nurse leaders describe the digital competencies and associated practices required to sustain PFCC within technology-mediated pediatric care?

### 1.3. Objectives

1. To describe the digital competencies that pediatric nurse leaders identify as essential for sustaining PFCC in technology-mediated pediatric practice.
2. To explore organizational, technological, and relational barriers and facilitators influencing the development and enactment of these competencies.
3. To examine how leaders understand the relationship between competency enactment in technology-mediated practice (e.g., documentation, communication, decision support, telehealth/portal use) and PFCC processes (dignity and respect, information sharing, participation, and collaboration).
4. To propose an empirically grounded, practice-oriented interpretive model mapping digitally competent pediatric nursing leadership for sustaining PFCC, derived from leaders’ lived experiences.

Using Interpretative Phenomenological Analysis (IPA), this study examines how pediatric nurse leaders make sense of digital leadership in technology-mediated care environments and generates an interpretive, practice-oriented competency map that can inform leadership development, guide curriculum design, and support organizational decisions on digital implementation while preserving PFCC. The following methodological approach was designed to capture the depth and nuance of leaders’ experiences within this evolving practice context.

## 2. Materials and Methods

### 2.1. Research Design

This study used Interpretative Phenomenological Analysis (IPA) to examine how pediatric nurse leaders make sense of digital leadership and the digital competencies required to sustain Patient- and Family-Centered Care (PFCC) [46]. IPA was selected for its idiographic commitment to detailed case analysis and its focus on interpretative meaning-making, supporting close examination of how leaders understand and enact digital work within the relational complexity of pediatric care. The study was informed by Heideggerian hermeneutic phenomenology, recognizing that experience is interpreted through professional, cultural, and technological horizons and that meaning is co-constructed through engagement with participants’ accounts while remaining grounded in those accounts [47]. Interpretation was theoretically contextualized only after idiographic analysis, to situate meanings within relevant scholarship without overriding participants’ lived experience or imposing an a priori framework [48,49]. The analytic stance prioritized phenomenological primacy throughout, with interpretative claims repeatedly checked against primary extracts.

### 2.2. Settings

The study was conducted across three hospitals in a major metropolitan region of Saudi Arabia, purposively selected from a sampling frame of 12 pediatric facilities, using maximum variation sampling to reflect differences in governance (public versus private), clinical acuity, service mix, and relative digital maturity. To preserve anonymity during peer review, sites are presented as Public Tertiary A, Public General B, and Private Tertiary C. Digital maturity was operationalized a priori for sampling purposes as the presence of: (1) a certified EHR with computerized provider order entry (CPOE) and electronic medication administration record (eMAR); (2) active pediatric telehealth; (3) a patient/family portal with ongoing enrollment workflows; (4) embedded clinical decision support (e.g., medication safety alerts); and (5) device integration (e.g., bedside monitors/NICU equipment) with secure clinical messaging. These indicators were used pragmatically to characterize relative maturity for maximum-variation sampling rather than as a formal maturity score. Relative maturity classifications were confirmed through review with nursing and informatics leadership and available site documentation.

### 2.3. Participants and Sampling

A purposive, maximum-variation sample of ten pediatric nurse leaders (N = 10) was recruited to ensure heterogeneity in leadership tier, unit type, years in leadership, and self-rated digital proficiency. Consistent with IPA, the sample size was determined by the attainment of idiographic depth within individual cases and the stabilization of cross-case patterning, rather than by statistical representativeness or grounded theory saturation [50]. By Cases 7 and 8, the cross-case thematic architecture had stabilized, and new cases contributed refinement and nuance rather than structurally new themes, consistent with the concept of informational sufficiency as described in current IPA methodological literature (Smith et al., 2009 [51]; Wanat et al., 2025 [52]). Cases 9 and 10 were retained to conduct systematic deviant and disconfirming case analysis, which tested and refined thematic boundaries. Furthermore, the dual-method approach—two in-depth interviews plus a four-week reflective journaling period—substantially enriched each participant’s dataset beyond what single-session interviews typically yield, strengthening the overall evidentiary basis. The ≥2-year pediatric leadership experience criterion was established to ensure that participants had experienced at least one substantive cycle of digital system implementation or significant upgrade (such as an electronic health record version transition or telehealth launch), providing the experiential depth necessary for IPA’s characteristic phenomenological and interpretive reflection.

Inclusion criteria: registered nurses in formal pediatric/neonatal leadership roles; ≥2 years of pediatric leadership experience; direct involvement in technology implementation and/or optimization initiatives; ongoing clinical responsibilities; Arabic or English fluency; and willingness to complete two interviews and the journaling protocol. Exclusion criteria: purely administrative roles without patient contact; temporary/acting positions; or inability to complete study procedures.

Recruitment was facilitated by nursing leaders who circulated study invitations; however, participation was opt-in, and initial contact was made directly with the Principal Investigator (PI) to preserve voluntariness. To reduce gatekeeper influence, recruitment proceeded within predefined strata based on leadership tier (unit/ward leader, department leader, and broader service/administrative leadership) and clinical area (e.g., NICU, inpatient pediatrics, and pediatric specialty/outpatient services), using a rotating approach across strata.

### 2.4. Data Collection

#### 2.4.1. Semi-Structured Interviews

Each participant completed two in-depth semi-structured interviews (60–90 min), scheduled approximately four weeks apart (3–5 weeks). The interview guide was developed through a focused review of the literature on digital competency, nursing leadership, and IPA-informed interview design; iterative drafting with reflexive review by the principal investigator; and pilot testing with two non-participating nurse leaders, which resulted in reordering of two question blocks and clarifying one probe that participants found ambiguous (pilot data excluded from analysis). Interview 1 explored leadership trajectory and meanings attached to digital leadership. Interview 2 drew on the participant’s journal to probe concrete episodes of digital competency enactment and PFCC-related tensions. Probing questions used throughout both interviews included: “Can you tell me more about that experience?”; “What were you thinking in that moment?”; “How did the family respond?”,” What did you do next, and why?”; “Was there anything that surprised you?”; and “How does that compare with what you described in your journal?” These probes illustrate the conversational, exploratory style consistent with IPA practice. The complete interview guide is provided as Supplementary Material S1. Interviews were conducted in private rooms within participating hospitals, in Arabic or English according to participants’ preference, audio-recorded, and accompanied by field notes. Across the sample, 20 interviews were conducted: 7 participants preferred Arabic (14 interviews) and 3 preferred English (6 interviews).

#### 2.4.2. Reflective Journaling

Between interviews, participants completed a four-week reflective journaling protocol using a secure, institution-approved platform [53]. Weekly prompts progressed from descriptive to analytic to integrative reflection. Entries were submitted as typed text, voice notes, or photographs of handwritten pages. Participants were instructed to omit identifying patient/family details and to avoid staff names or unit identifiers. Weekly reminders and optional check-ins supported completion [54].

#### 2.4.3. Data Integration

For each participant, interviews and journal entries were treated as a single idiographic corpus. Journal data informed, but did not constrain, the second interview. During analysis, an interpretive mapping matrix was used to track how meanings related across sources such as convergence, complementarity, dissonance, or silence, supporting temporal depth and cross-source coherence. This matrix was used as an interpretive tool rather than as a validity test and did not displace phenomenological primacy [55,56].

#### 2.4.4. Transcription and Translation

Transcripts were produced verbatim. Arabic interviews were translated into English by a certified medical translator. A purposive 30% sample, ensuring representation across sites and leadership tiers, was back-translated to assess fidelity. A bilingual advisory panel (n = 2) reviewed discrepancies to support semantic, conceptual, and experiential equivalence; translation decisions and rationales were documented in the audit trail. Where quotations originated in Arabic, the final English wording was reviewed in light of the translator’s notes and bilingual input to preserve meaning [57].

### 2.5. Procedure

**Figure 1:**
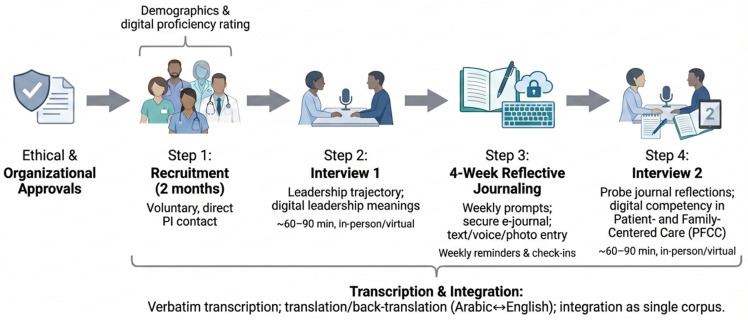
Timeline of data collection and integration procedures (two-interview and four-week journaling design).

Following ethical and organizational approvals, recruitment occurred over two months. Interested individuals contacted the PI directly to preserve the voluntariness of their participation. Participants completed a demographic form and a self-rating of digital proficiency. Data collection spanned approximately five months overall and followed the sequence shown in Figure 1.

### 2.6. Rigor, Reflexivity, and Interpretative Engagement

Rigor was guided by Yardley’s four principles. Sensitivity to context was addressed by situating accounts within Saudi healthcare structures, culturally situated perspectives on care and technology, and potential gendered dynamics in interviewing; translation decisions (Arabic ↔ English) were treated as interpretative acts and documented [47]. The principal investigator’s (PI) identity as a pediatric nurse educator and faculty member embedded in the same regional healthcare system was addressed explicitly through reflexive bracketing. The PI’s professional familiarity likely facilitated rapport and candor from participant leaders, while simultaneously requiring active documentation of normative assumptions about “correct” digital practice that could shape interpretive moves. Specific moments when educator-identity assumptions were tested or challenged were recorded in detailed reflexive memos. A second qualitative researcher with IPA expertise independently reviewed the analytic memos and cross-case theme structures, providing an external check on interpretive moves and strengthening collaborative validity. Commitment and rigor were supported through prolonged engagement (two interviews plus a four-week journaling period), idiographic depth, and systematic documentation of analytic decisions (reflexive memos, decision logs, and versioned codebooks).

Impact and importance were pursued by prioritizing practice-relevant insight into digital competencies in pediatric leadership. Reporting followed the COREQ (32-item) checklist [58] (Supplementary Material S2). Reflexivity permeated all phases. The PI (pediatric nurse educator) maintained a reflexive journal to document positionality, expectations about technology, and emotional responses, and practiced reflexive attending to assumptions to reduce premature closure during interpretation. Credibility and interpretative challenge were supported through iterative case-by-case analysis, reflexive memoing, a transparent audit trail of coding and theme decisions, and systematic searching for disconfirming evidence across interviews and journals, consistent with IPA’s emphasis on depth and interpretative accountability [59].

### 2.7. Data Analysis

Data analysis followed IPA as described by Smith, Flowers, and Larkin (2009), which was conducted in NVivo 14, with a maintained audit trail (analytic memos, decision logs, codebook iterations, and translation notes) [51]. For each case, interviews and journals were treated as a single idiographic corpus.

- Phase 1—Idiographic, phenomenological openness

Each case began with immersive reading and listening (audio transcript checks), followed by initial noting at three levels: (a) descriptive (events, objects of concern), (b) linguistic (metaphor, tone, hesitations), and (c) conceptual (tentative sense-making). Emergent themes were then crafted, retaining participants’ language where possible, and clustered using established IPA techniques (abstraction, subsumption, contextualization, and polarization). Numeration was used cautiously and only to support descriptive clarity. A within-case structure (superordinate and subordinate themes with exemplar extracts) was finalized before cross-case comparison. Deviant and disconfirming material was retained to test and refine thematic boundaries [52].

- Phase 2—Cross-case patterning

After idiographic closure for all cases, convergences and divergences across participants were examined, producing a master table of themes with definitions, illustrative quotations, and descriptive indication of breadth (i.e., how widely a theme appeared across cases). Contextual attributes (e.g., leadership tier, unit type, and site digital maturity) were used as sensitizing backdrops to interpret how meanings traveled, not to test associations.

- Phase 3—Theoretical contextualization (post-idiographic)

Selective theoretical lenses (e.g., Rogers’ Diffusion of Innovation; Benner’s novice-to-expert) were engaged only after themes stabilized to situate interpretations within broader discourse without determining them. Where theoretical framing risked overreach, interpretation returned to primary extracts to preserve phenomenological primacy [60].

- Cross-source integration (interviews and journals)

Cross-source integration used the interpretive mapping described in Section 2.4.3 to track convergence, complementarity, dissonance, and silence. Journals frequently captured temporal shifts (e.g., evolving recognition of family digital literacy barriers) and traced them back to interview narratives to deepen temporal texture without subordinating either source.

- Language and translation in analysis

Arabic interviews were coded in Arabic by the primary analyst; English translations supported team discussion. When interpretive pressure points arose (e.g., metaphors or culture-bound terms), audio recordings, translator notes, and translation memos were revisited to preserve semantic and experiential equivalence. Quotations reported in the Section 3 were lightly edited for readability while retaining meaning [61].

- Trustworthiness procedures

Interpretative moves were documented in analytic memos, and decisions altering codes/themes were logged in a structured audit trail. Theme boundaries were continuously tested against the full dataset (interviews and journals), with attention to negative cases and contextual nuance [62].

### 2.8. Ethical Considerations and Approval

Ethical approval was obtained from the relevant institutional review board and the research committees of all participating hospitals (Reference: KAU-IRB-2025-NREC-1F-177). The study was conducted in accordance with the Declaration of Helsinki and applicable national research governance requirements. Informed consent emphasized voluntariness, confidentiality through pseudonymization and encrypted storage, a five-year retention period, and the right to withdraw at any time without consequences. Transcripts were de-identified during transcription and translation, and potentially identifying contextual details were generalized or omitted to reduce the risk of deductive disclosure. To mitigate power dynamics in clinical hierarchies, interviews were conducted in private, neutral locations; participation was managed directly by the PI; and participants were assured that participation or non-participation would not affect professional standing. Cultural accommodation included gender-concordant interviewing upon request and scheduling around prayer times. Participants received certificates of participation without monetary compensation. Information about available support services was provided in case participation elicits discomfort. Access to raw data was restricted through role-based permissions on encrypted institutional servers, and data governance requirements were met to enable secure, anonymized collaboration through compliant data-sharing procedures.

## 3. Results

### 3.1. Overview of the Participants

Ten pediatric nurse leaders (N = 10) completed the full study protocol, comprising two in-depth interviews per participant and a four-week reflective journal. Participants were recruited across three hospitals with differing relative digital maturity (Public Tertiary A, Public General B, and Private Tertiary C). The sample comprised 8 women and 2 men aged 28–54 years, with 5–26 years of pediatric nursing experience and 2–15 years of leadership experience. Leadership roles represented multiple tiers, including charge nurses (n = 4), unit managers (n = 3), clinical nurse specialists (n = 2), and one nursing director (n = 1). Participants’ accounts frequently positioned digital systems as both enabling and potentially distancing, captured in remarks such as: “The screen can help us, but the family must still feel seen” (P03) and “Technology is my ally, not my boss” (P08). Table 1 summarizes participant characteristics.

### 3.2. Idiographic Vignettes (Illustrating Lived Experience)

To honor IPA’s idiographic commitment, we present three brief vignettes that illustrate how meanings were formed, shifted, and revised within individual participants’ accounts across interviews and journaling, before proceeding to cross-case synthesis. These vignettes fulfill a specific analytic function: they demonstrate the interpretive process of individual meaning-making rather than simply asserting thematic patterns. P03, P06, and P08 were selected because they represent the full range of leadership tiers in the study (charge nurse, unit manager, and clinical nurse specialist, respectively), the three study sites and their varying digital maturity levels (public tertiary, public general, and private tertiary), and they displayed especially transparent temporal meaning shifts in their journal entries—making them analytically productive windows into the phenomenon under study. Each vignette closes with an explicit connection to the cross-case theme(s) it prefigures, enabling readers to trace the analytic pathway from individual case to cross-case synthesis in subsequent sections.

#### 3.2.1. Case Vignette 1: P03 (Charge Nurse, Public Tertiary A)

“At first, the laptop was a wall. I felt I had to hide behind it to finish the orders… I’d be typing away, feeling like I was being rude but not knowing what else to do”.

(Interview 1)

“Lately, I turn it so the mother sees with me. I say, ‘This is your child’s plan, shall we check it together?’… the mother can see what I’m seeing, and she can ask questions right away”.

(Interview 1)

“Week 3: It’s not about pretending the screen isn’t there. It’s part of our dance—eyes to child, to mother, to screen, back again”.

(Journal)

Across sources, technology moved from “wall” to “dance,” with co-viewing and narrated actions emerging as practical strategies for sustaining relational presence while completing digital tasks.

#### 3.2.2. Case Vignette 2: P06 (Unit Manager, Public General B)

“Barcode failed again during a double-check. The alert was loud, the mother looked afraid… I told my junior, ‘Breathe first… the machine shouts, but the baby is quiet—listen to the quiet’”.

(Interview 2)

“Week 1: I blamed the nurse for a missed alert… Week 4: I posted my own mistake… Staff said, ‘We can say it now’”.

(Journal)

Digital safety mechanisms were experienced as both protective and disruptive. Leadership meaning-making involved buffering families from alarm intensity while using vulnerability to shift team culture from blame toward shared learning.

#### 3.2.3. Case Vignette 3: P08 (Clinical Nurse Specialist, Private Tertiary C)

“The portal helps, but some parents don’t use it… I tell them, ‘You can read later, but ask me now.’ For Arabic-only families, we add a voice note—they hear warmth”.

(Interview 1)

“Week 2: Family angry after getting lab results on the portal first… Week 3: New rule—no bad news by portal”.

(Journal)

Here, “warmth” functioned as a culturally legible marker of caring presence, prompting multimodal communication and new boundaries for sensitive disclosure.

### 3.3. Cross-Case Patterning: Superordinate Themes and Subthemes

After completing idiographic analyses for each participant, cross-case patterning yielded four superordinate themes, each with related subthemes (Table 2). Counts are provided only to indicate breadth across cases and to increase transparency; they are not claims of prevalence or statistical inference.

The two subthemes within each superordinate theme are constitutively related: together they enact the overarching competency domain rather than representing independent skills. For Theme 1, gaze choreography and narrated action together constitute the management of embodied presence across digital–human boundaries; neither alone is sufficient, as presence requires both spatial positioning and relational narration. For Theme 2, micro-coaching and normalized near-miss disclosure together constitute vulnerable expertise: micro-coaching enacts competence in the moment, while disclosure reconstructs authority through transparency over time. For Theme 3, language scaffolding and portal boundary-setting together constitute culturally legible communication; one addresses real-time comprehension, the other manages the conditions under which digital communication is appropriate and timely. For Theme 4, override documentation and integration of system indicators with family cues together constitute calibrated judgment: documentation makes clinical reasoning auditable, while integration ensures that algorithmic prompts serve rather than override situational and relational knowledge.

#### 3.3.1. Theme 1: Negotiating Embodied Presence Across Digital–Human Boundaries

1a. Choreographing gaze and screen position. Participants described deliberate management of gaze, posture, and device placement to avoid families experiencing the EHR as a barrier. “I turn the laptop, so the mother sees what I’m checking”.

(P03)

1b. Narrating actions to keep families “in the room.” Narration functioned as a relational bridge when attention was drawn to the screen: “I speak my steps… When I don’t narrate, the room goes cold”.

(P01)

#### 3.3.2. Theme 2: Leading Through Vulnerable Expertise Amid Digital Uncertainty

2a. Micro-coaching at the point of care. Leaders described rapid, bedside coaching that translated alerts into patient-oriented reasoning: “Two questions in thirty seconds: ‘What does the alert want? What does the child need?’”.

(P04)

2b. Normalizing disclosure of near-misses and errors. Several leaders described using their own fallibility to open safer discussion about technology-related slips: “I posted my own error; after that, near-miss reports came with less shaking hands”.

(P06)

#### 3.3.3. Theme 3: Crafting Culturally Legible Digital Communication

3a. Language and digital-literacy scaffolding. Participants used multimodality (e.g., voice notes, teach-back) to support understanding while preserving a sense of caring presence: “Portal link plus a voice note in Arabic—then I ask them to show me back”.

(P05)

3b. Setting boundaries for sensitive content in portals/telehealth. Participants described boundary rules that protected dignity during emotionally charged communication: “No bad news by portal. Same-day call instead”.

(P08)

#### 3.3.4. Theme 4: Calibrating Clinical Judgment with Data and Decision Support

4a. Documenting rationales for corrections/overrides. Leaders emphasized making clinical judgment auditable and teachable within the record: “The allergy flag was wrong… we corrected it and wrote why”.

(P04)

4b. Integrating system indicators with family and bedside cues. Participants described using digital indicators as supportive inputs while prioritizing family needs and clinical cues: “The system helps me plan, but I still add time for the anxious family”.

(P03)

### 3.4. Cross-Source Relationships and Lived Tensions (Interviews + Journals)

Consistent with the planned integration approach, interviews and journals were treated as one idiographic corpus, and cross-source relationships were mapped as convergence, complementarity, dissonance, or silence. Across cases, several convergences were prominent: co-viewing and narration to preserve presence; point-of-care micro-coaching; layered communication aligned with language and digital literacy; and concise documentation of rationales when correcting or overriding system prompts. These tensions were not treated as analytic noise but as the very substance of leadership judgment in action, each tension representing a moment in which competency was actively and visibly enacted rather than merely exercised as background skill. Deviant case analysis strengthened thematic boundaries: P09, for example, expressed ambivalence about vulnerability disclosure, worrying that visible uncertainty could undermine perceived competence. This disconfirming case prompted refinement of the “vulnerable expertise” theme, clarifying that the efficacy of vulnerability disclosure was contingent on established relational trust and on the organizational safety culture within the unit, a boundary condition not initially evident from the convergent cases.

Tensions were also evident and were interpreted as leadership judgments enacted in real time:

- Transparency vs. preparation: “Families should see everything we see” (P01) contrasted with accounts emphasizing pacing and explanation before sharing screens (P07).
- Safety vs. affect: Alerts were experienced as protective yet capable of provoking fear; leaders buffered families by pausing, explaining, and re-checking steps (P06).
- Vulnerability vs. perceived competence: Some leaders viewed error disclosure as enabling learning, while others worried it could be misread as incompetence (P09).
- System guidance vs. situational need: Decision support was treated as informative, yet bedside cues and family context frequently shaped final priorities (P03; P06).

### 3.5. Temporal Movements in Meaning-Making (Journals)

Reflective journaling captured meaning shifts over time, often following practical disruptions (e.g., alert events, portal-mediated disclosures) that precipitated changes in leadership practices. Examples included:

P03: “Laptop was a wall” evolving to “part of our dance,” alongside adoption of co-viewing and narrated actions.

P06: Movement from a blame response following a missed alert to leader self-disclosure and a more open reporting climate.

P08: A portal-first disclosure conflict prompting a team boundary (“no bad news by portal”).

Across multiple journals, participants described iterative adjustment of routines (e.g., screen positioning, narration, multimodal explanation) as they sought to align digital work with PFCC goals.

### 3.6. Analytic Visibility and Interpretative Accountability

Idiographic analysis was conducted case by case using descriptive, linguistic, and conceptual noting, followed by within-case clustering before cross-case patterning. Metaphors and embodied language (e.g., “wall,” “dance,” “warmth,” “cold room”) were treated as meaning-bearing and traced across interviews and journals. Breadth indicators (e.g., “9/10”) are reported only to show distribution across cases and enhance transparency; they do not imply quantification of experience. Deviant instances (e.g., limiting co-viewing for sensitive discussions) were retained to refine thematic boundaries. Arabic data were coded in Arabic, and translation notes were used to preserve semantic and experiential meaning in reported quotations.

### 3.7. Integrative Output: Interpretive Model of Digital Competencies for Sustaining PFCC

**Figure 2:**
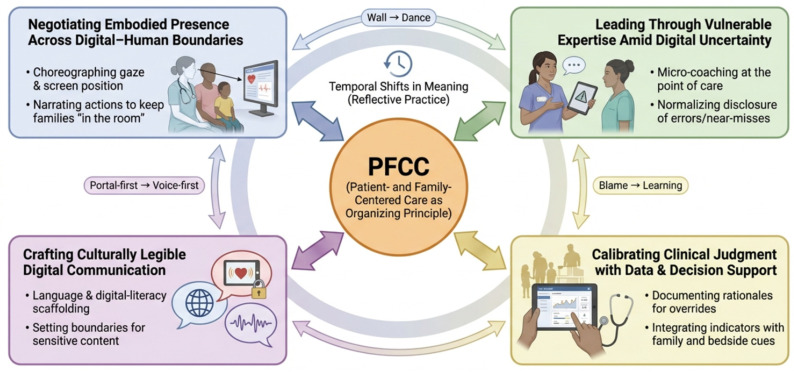
Interpretive model of digital competencies for pediatric nurse leadership sustaining PFCC in technology-mediated care.

The four superordinate themes are presented as interconnected processes constituting an interpretive model of digital competencies for pediatric nurse leadership in PFCC-oriented, technology-mediated care (Figure 2). This model is not a simple summary of four independent themes; rather, it is a relational, practice-embedded architecture specifying how the four competency domains are dynamically interrelated and mutually reinforcing, organized around PFCC as the governing value. Specifically, culturally legible communication is both a prerequisite for and a product of relational digital presence: leaders must first establish cultural and linguistic alignment before co-viewing is experienced as inclusive rather than intrusive, and co-viewing, in turn, generates new opportunities for culturally responsive explanation. Calibrated judgment with data requires vulnerable expertise to be enacted safely: leaders who model transparency about system uncertainty create the psychological conditions under which staff are willing to question algorithmic prompts and prioritize family-contextual cues. All four domains are simultaneously activated when navigating complex family encounters.

PFCC functions not merely as the center of the diagram but as the organizing principle that gives directionality and ethical coherence to each enactment of competency; each practice described in the themes is oriented toward the question of how digital action can preserve or strengthen the family’s meaningful partnership in care. The model’s status is explicitly contextual: it is a contextually grounded interpretive framework derived from the lived experiences of ten pediatric nurse leaders in a specific institutional context in Saudi Arabia, warranting empirical evaluation and interpretive testing before application or generalization to other cultural, organizational, or technological settings. Temporal shifts documented in journals (e.g., barrier → shared choreography; blame → learning-oriented vulnerability; portal-first → voice-first boundary setting) further illustrate that competency enactment evolved through practice-based meaning-making rather than static skill acquisition.

Figure 2 shows the interpretive model of digital competencies for pediatric nurse leadership to sustain PFCC. The model was derived from an Interpretative Phenomenological Analysis of 10 pediatric nurse leaders across three hospitals with differing levels of relative digital maturity (Public Tertiary A, Public General B, Private Tertiary C). PFCC is positioned as the organizing principle for enacting competencies. Bidirectional arrows represent dynamic interrelationships, and the temporal element reflects meaning shifts identified through reflective journaling.

## 4. Discussion

This interpretative phenomenological analysis (IPA) addressed the research question by illuminating how pediatric nurse leaders make sense of the enactment of digital competency while sustaining patient- and family-centered care (PFCC) in technology-mediated practice. Across participants’ accounts and reflective journals, digital competence was experienced not as technical proficiency alone, but as an integrated set of relational, culturally situated, and clinically grounded practices that leaders continually refined as digital systems entered the encounter [63]. The interpretive model derived from the analysis specifies four interlinked competency domains—negotiating embodied presence across digital–human boundaries, leading through vulnerable expertise amid digital uncertainty, crafting culturally legible digital communication, and calibrating clinical judgment with data and decision support—organized around PFCC as the guiding value. The following discussion explicitly positions each domain relative to prior literature: what is new, what extends or confirms prior knowledge, and where findings are in tension with existing frameworks. Findings emerged from participants’ own language, metaphors, and enacted practices (e.g., “wall,” “dance,” “warmth” as analytic anchor points) through IPA’s idiographic close reading, within-case theme crafting, cross-case comparison, and post-idiographic theoretical contextualization—rather than from a priori categorization.

### 4.1. Digital Competence as Relational Enactment Rather than Technical Mastery

A central contribution of this study is the finding that leaders did not describe digital competence as “using systems correctly” in isolation; rather, competence was constituted through how digital work was made relationally and ethically intelligible to families. Participants’ accounts demonstrated that the clinical encounter is shaped by where attention goes and how it is redistributed when documentation and decision support are present. Therefore, leaders treated embodied practices, screen positioning, co-viewing, and narrating actions as core competency elements because these practices actively preserve dignity, respect, and collaboration by keeping families oriented to what is happening and why. The shift described in journals from “technology as a wall” to “technology as part of our dance” illustrates how leaders’ meaning-making evolved through lived experience: digital tools were reinterpreted from barriers to shared objects that can support partnership when interactions are intentionally choreographed. Importantly, this reframing did not deny the disruptive potential of digital systems; instead, it specified the micro-practices through which leaders counteracted relational drift and maintained PFCC during screen-mediated work.

### 4.2. “Vulnerable Expertise” and the Reconstruction of Authority in Digital Uncertainty

The theme of leading through vulnerable expertise highlights an under-recognized dimension of digital leadership: authority was frequently enacted through transparency, coaching, and shared learning rather than through uninterrupted certainty. “Vulnerable expertise” is here defined as a leadership stance in which authority is enacted through transparent acknowledgment of digital uncertainty, the pedagogical use of personal fallibility, and sustained relational accountability to families and staff, rather than through the projection of uninterrupted technical certainty. This construct is novel in the nursing digital competency literature, building on Edmondson’s psychological safety work and Mertens et al.’s (2025) research on leader humility in nursing [64], but applying these constructs specifically to technology-mediated uncertainty in pediatric care. Existing competency frameworks that position digital competence as primarily technical proficiency are inconsistent with this relational and interpretive enactment; the relational dimension described by participants in this study was not captured by any of the three frameworks reviewed (DigComp, TIGER, AONL), representing a genuinely new specification rather than an extension of prior work. Participants described digital environments as inherently uncertain, alerts may be inaccurate, interfaces may fail, and new workflows may introduce novel risks requiring leaders to guide staff in interpreting technology without becoming dominated by it. Openly acknowledging fallibility was portrayed as a means of reducing fear and strengthening psychological safety, enabling near-misses and errors to be discussed as learning opportunities [65]. These accounts suggest that “confidence” in digital leadership may be better conceptualized not only as personal assurance, but also as the capacity to remain grounded, accountable, and relationally steady in the face of digital uncertainty [64].

### 4.3. Cultural Legibility and Equity as Practical Leadership Work

Leaders’ accounts also show that digital competence is culturally and linguistically situated. “Culturally legible communication” is defined here as the practice of calibrating digital communication modalities, including format, language, timing, medium, and pacing, to align with the family’s cultural, linguistic, and health literacy profile, ensuring that information is not merely transmitted but meaningfully received, understood, and actionable [66]. This extends the digital equity literature to leadership enactment in a Middle Eastern, Arabic-language context, a perspective rarely represented in the existing digital nursing leadership literature. Participants consistently described multimodal adaptations (Arabic voice notes, visual summaries, staged disclosure) that were not prescribed by institutional protocols but arose from leaders’ attunement to individual family needs, reinforcing that cultural legibility is enacted rather than instructed [67,68,69].

### 4.4. Judgment with Data and Decision Support: Keeping PFCC as the Organizing Principle

Finally, participants’ meaning-making clarified how leaders balanced data-driven prompts with situational, family-centered judgment. Digital indicators and decision support were treated as valuable inputs that could improve coordination and safety, yet leaders emphasized that these signals do not resolve the moral and relational dimensions of care. Competency was enacted through calibration: documenting rationales for corrections/overrides to preserve accountability and teachability, while integrating digital cues with bedside assessment and family needs. In this way, PFCC remained the organizing principle that governed when and how technology should guide action. This theme is particularly relevant to contemporary pediatric environments, where decision support and safety alerts can become interactionally dominant; leaders’ accounts indicate that human judgment and relational presence can coexist with digital guidance without succumbing to technological determinism [70].

### 4.5. Theoretical and Practical Implications

#### 4.5.1. Theoretical Implications

These findings extend current conceptualizations of digital competence by specifying how competence is enacted in the lived realities of pediatric leadership. First, the results suggest that digital competence in leadership is best understood as simultaneous and integrated across relational, cultural, and clinical domains rather than as a linear checklist of discrete skills. Second, the interpretive model positions PFCC as an organizing value that shapes digital enactment, highlighting that competence involves value-consistent decision-making as much as technical capability. Third, the analysis introduces “vulnerable expertise” as a meaningful leadership construct in digital transformation: authority is reconstructed through transparency, micro-coaching, and reflective learning in response to uncertainty. Together, these contributions indicate that existing competency frameworks and measures may require refinement to capture embodied presence, cultural meaning-making, and judgment-in-context as central components of digitally competent pediatric nursing leadership [71].

#### 4.5.2. Practical Implications

The interpretive model suggests actionable and potentially trainable practices that organizations could incorporate into leadership development and implementation efforts while recognizing that these practices require further observational and outcome-focused evaluation. At the encounter level, leaders can be trained in “screen-side” practices that preserve PFCC, including: (1) co-viewing and deliberate screen positioning; (2) narrating digital actions and using plain-language explanations to keep families oriented; (3) multimodal, language-congruent communication with teach-back; and (4) explicit boundaries for sensitive disclosures (e.g., avoiding portal-only communication for distressing results). At the team level, leaders can strengthen psychological safety by practicing micro-coaching at the point of care and modeling transparent learning when digital processes fail. At the system level, informatics and policy teams can enable these behaviors by designing EHR features that support shared viewing, reduce documentation friction during family encounters, provide simple fields for rationale documentation when correcting or overriding prompts, and offer guidance that supports culturally appropriate communication modalities. Importantly, these recommendations are intended as practice-oriented propositions derived from leaders’ self-reported lived experiences, rather than as evidence of observed practice change or demonstrated effects on patient, family, staff, or clinical outcomes; therefore, they warrant further evaluation across different settings using observational, multi-stakeholder, and outcome-sensitive designs [72].

### 4.6. Strengths and Limitations

#### 4.6.1. Strengths

This study’s methodological congruence with IPA enabled close examination of leaders’ meaning-making in ways not readily accessible to quantitative designs. The dual-interview structure, combined with four-week reflective journaling, provided temporal depth and captured shifts in interpretation and practice over time. Maximum-variation sampling across three sites with differing relative digital maturity enhanced transferability by broadening the contextual range. Rigorous translation and bilingual review procedures supported semantic and experiential equivalence across Arabic and English data and strengthened interpretive credibility.

#### 4.6.2. Limitations

As an idiographic qualitative study, the sample (N = 10) supports depth rather than experiential breadth; transferability, therefore, depends on contextual fit rather than statistical generalization. The single-country context may limit transferability to settings with different norms regarding family involvement, communication, and digital disclosure. The four-week journaling window may not capture longer-term competency development or organizational change trajectories. Participants were pediatric nurse leaders involved in implementation or optimization initiatives, which may introduce selection bias toward individuals with higher engagement, confidence, or interest in digital systems.

A further limitation is the study’s reliance on leaders’ self-reported accounts generated through interviews and reflective journals. Although this approach was methodologically congruent with interpretative phenomenological analysis and enabled close examination of meaning-making over time, it did not include direct observation of clinical encounters, independent assessment of digital competency enactment, or triangulation with staff nurses, children, families, physicians, informatics personnel, or clinical outcome data. Reflective journals provided temporal depth and allowed for within-participant comparison, but they should not be interpreted as full methodological triangulation because they remained participant-generated accounts. Accordingly, the findings should be understood as an interpretive account of how pediatric nurse leaders perceived, negotiated, and described digital competency enactment, rather than as evidence that these competencies were consistently observed in practice or that they improved patient- and family-centered care processes, safety indicators, family experience, or clinical outcomes. Future research should therefore examine whether these competency domains are observable in real encounters and whether they are associated with measurable outcomes such as family engagement, communication quality, digital safety practices, near-miss reporting, psychological safety, patient/family experience, or care quality indicators.

### 4.7. Recommendations and Future Research

#### 4.7.1. Practice Recommendations

Healthcare organizations should develop pediatric digital leadership programs that explicitly integrate PFCC into digital work. Training should move beyond technical “tool use” toward simulation and reflective learning focused on (a) maintaining embodied presence during documentation and decision support use, (b) transparently communicating digital actions to families, (c) delivering culturally and linguistically appropriate digital communication, and (d) calibrating judgment when digital prompts conflict with situational family needs. Structured debriefing and peer-mentoring processes can support “vulnerable expertise” by normalizing learning from digital disruptions and strengthening psychological safety.

#### 4.7.2. Future Research

Future studies should examine competency development longitudinally over extended periods to capture longer-term meaning-making, behavioral enactment, and organizational adaptation. Comparative research across cultural contexts would clarify which competencies are broadly transferable and which are culturally contingent. To address the limitations of self-reported data, future research should incorporate direct observation, ethnographic fieldwork, simulation-based assessment, or video-reflexive methods to examine whether and how the proposed competency domains are enacted in real pediatric care encounters. Multi-stakeholder studies involving staff nurses, children and families, physicians, informatics personnel, and nurse managers are also needed to assess whether these domains reflect shared experiences across the care system. Intervention and implementation studies should test training programs derived from the interpretive model and evaluate feasibility, acceptability, fidelity, implementation outcomes, and observable changes in practice. Finally, mixed-methods research could integrate interpretative phenomenological insights with carefully selected process and outcome indicators, such as family-reported communication experiences, partnership measures, digital safety practices, near-miss reporting, psychological safety, patient/family experience, and care quality indicators, to strengthen evidence on how digitally competent leadership may support patient- and family-centred care in technology-mediated pediatric settings.

## 5. Conclusions

This study clarifies how pediatric nurse leaders perceive and negotiate the enactment of digital competence to sustain patient- and family-centred care in technology-mediated pediatric settings. The interpretive model identifies four interlinked competency domains: negotiating embodied presence, enacting vulnerable expertise amid digital uncertainty, crafting culturally legible digital communication, and calibrating clinical judgment with data and decision support. Together, these domains position patient- and family-centred care as the guiding principle for digitally competent pediatric nursing leadership.

The findings offer practice-oriented propositions and potentially trainable micro-practices, including co-viewing, narrating digital actions, using culturally congruent multimodal communication, setting portal boundaries for sensitive content, and documenting rationales for corrections or overrides. These insights may inform leadership development, simulation curricula, and digital implementation strategies. However, because the study was situated within a single-country context and based on leaders’ self-reported accounts, the model should be interpreted as a contextually grounded account of perceived competency enactment rather than as evidence of observed practice change or clinical impact. Future observational, multi-stakeholder, implementation, and outcome-focused research is needed to examine how these competency domains are enacted in practice and whether they are associated with improvements in family engagement, communication quality, digital safety, patient/family experience, and care quality outcomes.

## Figures and Tables

**Table 1:** Demographic and Professional Characteristics of Study Participants (n = 10).

Characteristic	n or Median [Range]
Gender	Female 8; Male 2
Age (years)	39 [28,29,30,31,32,33,34,35,36,37,38,39,40,41,42,43,44,45,46,47,48,49,50,51,52,53,54]
Pediatric experience (years)	13 [5,6,7,8,9,10,11,12,13,14,15,16,17,18,19,20,21,22,23,24,25,26]
Leadership experience (years)	6 [2,3,4,5,6,7,8,9,10,11,12,13,14,15]
Role	Charge nurse 4; Unit manager 3; Clinical nurse specialist 2; Nursing director 1
Primary unit	General pediatrics 4; NICU 3; Subspecialty 3
Site	Public Tertiary A 4; Public General B 3; Private Tertiary C 3
Highest degree	BSN 5; MSN 3; PhD 2
Self-rated digital proficiency (1–5)	4 [3,4]
Involved in EHR rollout	7
Involved in telehealth implementation	6
Involved in device integration and/or decision support configuration	4

Note. NICU = neonatal intensive care unit; EHR = electronic health record. Values are counts or median [range]. Self-rated digital proficiency was assessed on a 1 (lowest) to 5 (highest) scale. All data are descriptive only; counts reflect individual participant characteristics and are not treated as prevalence estimates or statistical inferences.

**Table 2:** Superordinate Themes and Subthemes Derived from Cross-Case Patterning.

Superordinate Theme (Breadth Across Cases)	Subthemes (Breadth Across Cases)
Theme 1. Negotiating embodied presence across digital–human boundaries (10/10)	1a. Choreographing gaze and screen position (9/10) 1b. Narrating actions to keep families “in the room” (9/10)
Theme 2. Leading through vulnerable expertise amid digital uncertainty (10/10)	2a. Micro-coaching at the point of care (10/10) 2b. Normalizing disclosure of near-misses and errors (9/10)
Theme 3. Crafting culturally legible digital communication (9/10)	3a. Language and digital-literacy scaffolding (9/10) 3b. Setting boundaries for sensitive content in portals/telehealth (7/10)
Theme 4. Calibrating clinical judgment with data and decision support (9/10)	4a. Documenting rationales for corrections/overrides (8/10) 4b. Integrating system indicators with family and bedside cues (8/10)

Note. Breadth indicators (n/10) reflect the number of individual participants whose accounts contributed to each superordinate theme or subtheme during cross-case patterning. These figures represent descriptive distributions across the sample only and do not constitute prevalence estimates, frequency claims, or statistical inferences.

## Data Availability

Due to the qualitative nature of the dataset and the potential risk of participant identification, the interview and journal materials are not publicly available. De-identified excerpts supporting the findings may be provided by the author upon reasonable request, subject to ethics approvals and a data-use agreement.

## Supplementary Materials

The following supporting information can be downloaded at: https://www.mdpi.com/article/10.3390/healthcare14101303/s1, Supplementary Material S1: Interview Guide and Journaling Protocol. Supplementary Material S2: COREQ 32-Item Checklist [73].
