# Supplementary material for: Digital Competencies for Pediatric Nurse Leaders to Sustain Patient- and Family-Centered Care: An Interpretative Phenomenological Analysis
Source: Healthcare (Basel). 2026 May 11;14(10):1303. doi: 10.3390/healthcare14101303 (PMC13205459; doi:10.3390/healthcare14101303)
Supplement: Supplementary file 1 [file healthcare-14-01303-s001.zip › healthcare-4161289-supplementary 1.pdf]

SUPPLEMENTARY MATERIAL S1

Semi-Structured Interview Guide and Reflective Journaling Protocol

Digital Competencies for Pediatric Nurse Leaders to Sustain Patient- and Family-Centered Care: An Interpretative Phenomenological Analysis

Alaa Hussain Hafiz | Department of Maternity and Child Health Nursing, King Abdulaziz University

Overview. This guide was used across two in-depth semi-structured interviews (60–90 min each) conducted approximately four weeks apart, and a four-week reflective journaling protocol. The guide was developed through a focused literature review on digital competency, nursing leadership, and IPA-informed interview design; iterative drafting with reflexive review by the principal investigator (PI); and pilot testing with two non-participating nurse leaders, which resulted in reordering two question blocks and clarifying one probe found to be ambiguous. Pilot data were excluded from the analysis. Interview 1 explored leadership trajectory and meanings attached to digital leadership. Interview 2 drew on participants' journal entries to probe concrete episodes of digital competency enactment and tensions with Patient- and Family-Centered Care (PFCC). All interviews were conducted in Arabic or English according to participants' preference, audio-recorded, and accompanied by the researcher's field notes.

Section 1. General Interview Procedures

The following procedural steps were followed at the opening of each interview:

- Confirm voluntary participation and right to withdraw without consequence.
- Obtain (or re-confirm) audio-recording consent.
- Confirm preferred language (Arabic or English) for the session.
- Remind the participant that there are no right or wrong answers; the focus is on their personal experience and meaning-making.
- For Interview 2 only: briefly review key themes from Interview 1 and journal entries to orient the conversation.

Probing questions used throughout both interviews included: "Can you tell me more about that?"; "What were you thinking in that moment?"; "How did the family respond?"; "What did you do next, and why?"; "Was there anything that surprised you?"; and (Interview 2 only) "How does that compare with what you described in your journal?"

Section 2. Interview 1 — Leadership Trajectory and Meanings of Digital Leadership

Duration: 60–90 minutes. Focus: biographical and professional grounding; participants' subjective meanings of digital leadership and PFCC; initial orientations to technology in their practice.

Part A. Opening and Professional Context

1. Could you start by telling me about your nursing career and how you came to be in your current leadership role in pediatrics?
   - *What drew you specifically to pediatric nursing?*
   - *How has your role evolved over the years, particularly in relation to digital technologies?*
2. How would you describe your unit or setting in terms of how digitized or technology-dependent it is?
   - *What digital systems are you working with day-to-day?*
   - *How long have these systems been in place? Were you involved in their introduction?*
3. When you hear the phrase "digital leadership" in nursing, what does that bring to mind for you?
   - *Is that different from how you thought about leadership before technology became so central?*
   - *Do you see yourself as a "digital leader"? In what sense?*

Part B. Digital Systems and Patient- and Family-Centered Care

4. How do you see the relationship between the digital systems in your unit and your ability to provide care that is truly centered on the patient and family?
   - *Has digitization made it easier or harder to involve families in care? Can you give an example?*
   - *Are there moments when technology seems to support PFCC, and moments when it seems to get in the way?*
5. Can you describe a situation—perhaps a memorable one—when you noticed that technology was affecting how a family experienced care, either positively or negatively?
   - *What was happening? Who was there?*
   - *What did you do as a leader in that moment, and why?*
   - *Looking back, what would you do differently, if anything?*
6. In your experience, what do pediatric nurse leaders need to be able to do—digitally—in order to keep the family truly at the center of care?
   - *Are there specific skills, behaviors, or mindsets you have developed or seen in colleagues?*
   - *How did you develop those? Through training, experience, observation of others?*

Part C. Technology, Communication, and Family Presence

7. When you are in a family encounter and also have to document or respond to system alerts, how do you manage that?

- *Do you have particular habits or strategies for keeping families engaged while using the EHR?*
- *How do you think families perceive you when your attention is partly on a screen?*

8. How do families in your unit engage with digital tools such as patient portals, telehealth, or electronic information?
   - *Are there families who seem less comfortable or less able to use these tools?*
   - *How does that affect the care you provide or the way you communicate with them?*
9. Are there cultural or language-related aspects of digital communication that are particularly salient in your setting?
   - *How do you adapt your communication when families have different languages or levels of digital literacy?*
   - *Have you developed any practices specifically for Arabic-speaking families or families new to digital health tools?*

Part D. Decision Support, Safety, and Clinical Judgment

10. How do you use clinical decision support tools or alerts in your practice, and how do you help staff use them?
    - *Can you describe a situation where an alert was helpful, and one where it created difficulty or uncertainty?*
    - *How do you decide when to follow an alert and when to use your own judgment?*
11. When a technology-related error or near-miss has occurred in your unit, how have you handled it as a leader?
    - *How did you communicate about it with staff? With families?*
    - *What did that experience teach you about leadership in a digital environment?*

Part E. Closing — Interview 1

12. As we come to the end of this conversation, is there anything you would like to add about what digital leadership means to you, or about the challenges and satisfactions of leading in a digital care environment?

Introduce the reflective journaling protocol (see Section 4). Confirm the platform, timeline, and format. Answer any questions.

Section 3. Interview 2 — Competency Enactment and PFCC Tensions in Technology-Mediated Practice

Duration: 60–90 minutes. Focus: concrete episodes drawn from journal entries; deeper exploration of competency enactment in specific digital contexts; tensions and resolutions; meaning shifts over time.

Preparation: Prior to Interview 2, the PI reviewed each participant's journal entries and developed 3–5 individualized probes based on specific episodes or reflections recorded. These are noted in each participant's case file and are introduced at appropriate moments within the guide structure below.

Part A. Opening and Journal Reflection

13. It has been about four weeks since we last spoke, and you have been keeping a journal during that time. How has that experience been for you?
    - *Were there entries that surprised you, or that you found yourself returning to?*
    - *Did the journaling change the way you thought about anything in your day-to-day practice?*
14. [Refer to a specific journal entry or theme.] In your journal, you described [episode/reflection]. Can you tell me more about what was happening and what you were thinking at the time?
    - *What did you do, and why did you make that choice?*
    - *How did the family respond? What did that mean to you?*
    - *How does what you wrote then compare with how you see it now?*

Part B. Deepening Competency Enactment

15. Looking back over the past four weeks, can you identify a moment when you felt you were at your most effective as a digital leader—when things came together well in terms of both technology and family care?
    - *What were the conditions that made that possible?*
    - *What specific actions did you take?*
16. And, conversely, was there a moment when digital leadership felt particularly difficult or when you felt pulled between technology demands and family-centered care?
    - *What was that like?*
    - *How did you resolve—or not resolve—that tension?*
    - *What did you learn from it?*
17. Has the way you position yourself, your body, or devices during family encounters changed at all since Interview 1? Has anything shifted?
    - *Can you describe what that looks like in practice?*
    - *Do you narrate your actions to families? If so, how do you do that?*

18. Since Interview 1, have there been any situations involving clinical decision support, alerts, or electronic documentation that you found particularly meaningful or challenging in relation to PFCC?
    - *How did you document your reasoning? Do you write rationales when you override or correct system recommendations?*
    - *How do you teach staff to handle these situations?*

Part C. Communication, Culture, and Boundaries

19. Have you used—or observed your team using—any specific communication strategies (such as voice notes, teach-back, or multimodal explanations) to support families with different language needs or digital literacy levels?
    - *What worked, and what did not?*
    - *Have you developed any team norms or informal policies around this?*
20. Since we last spoke, have there been any situations involving portal notifications, telehealth encounters, or digital messaging that raised questions about how and when to share sensitive information with families?
    - *How did you handle those situations?*
    - *Has your thinking about boundaries for sensitive digital communication shifted at all?*

Part D. Vulnerable Expertise and Team Culture

21. In your journal or since we last spoke, have you had opportunities to share with your team moments of uncertainty or mistakes involving digital systems?
    - *How did you approach that? How did staff respond?*
    - *In what ways, if any, do you think transparency about fallibility affects how your team works together or communicates with families?*
22. How do you support staff members who are less confident with digital systems or who struggle to maintain PFCC when technology is demanding?
    - *What does coaching in those moments look like?*
    - *Do you model particular behaviors or phrases that staff have taken up?*

Part E. Meaning-Making and Interpretive Synthesis

23. Looking across both of our conversations and your journal, how has your understanding of what it means to be a digitally competent pediatric nurse leader changed or deepened?
    - *What would you say to a colleague who was just taking on a leadership role in a digitally mature pediatric unit?*
    - *Is there a metaphor or image that captures how you now think about the relationship between technology and family-centered care?*

24. Is there anything we have not discussed that you think is important for understanding digital leadership and family-centered care in pediatric nursing?

Close: Thank the participant. Confirm data handling, confidentiality, and publication process. Provide information about available support services.

Section 4. Four-Week Reflective Journaling Protocol

Participants were provided with this protocol following Interview 1. Entries were submitted via a secure, institution-approved platform as typed text, voice notes, or photographs of handwritten pages. Participants were instructed to omit identifying patient and family details and to avoid using staff names or unit identifiers. Weekly reminders and optional brief check-ins with the PI were offered to support completion.

Instructions for Participants

Over the next four weeks, you are invited to keep a reflective journal about your experiences as a pediatric nurse leader working with digital technologies. There is no minimum or maximum length. You may write as much or as little as feels meaningful after each week. Your entries will remain confidential, will be used only within this research project, and will inform our second conversation. Please do not include patients' or families' names or any information that could identify them. Likewise, please avoid using the names of colleagues or identifying your unit directly.

Week 1: Descriptive Reflection

- *Describe two or three specific incidents this week in which a digital system (EHR, telehealth, portal, alert, device) played a role in a family encounter. What happened? Who was present? What did you do?*
- *How did you feel during and after each incident? What did you notice about the family's response?*
- *Was there a moment this week when technology seemed to support your relationship with a family, and a moment when it felt like an obstacle? Describe both.*

Week 2: Analytic Reflection

- *Looking back over last week, what tensions did you notice between managing digital systems and providing family-centered care?*
- *Was there a moment when you had to make a judgment call about whether to follow a system recommendation or trust your clinical and relational instincts? What did you decide, and why?*
- *How did you communicate with families about what was happening on the screen or in the documentation? What worked, and what felt incomplete?*

Week 3: Analytic Reflection: Leadership and Team

- *Were there moments this week when you coached or supported a staff member in navigating digital systems in a family encounter? What did you say or do?*

- *Did any technology-related errors, near-misses, or unexpected events occur? How did you handle them, and what did you communicate to staff and families?*
- *How are your team's digital practices supporting—or complicating—your goal of keeping families genuinely involved in care?*

Week 4: Integrative Reflection

- *Looking back across these four weeks, what has shifted in how you think about digital leadership and family-centered care?*
- *What does it mean to you now to be a 'digitally competent' pediatric nurse leader?*
- *If you were to describe the most important thing you have learned or recognized through this process, what would it be? Is there a phrase, image, or moment that captures it?*

Section 5. Participant Demographic and Digital Proficiency Form

Completed by participants prior to Interview 1. Used for descriptive characterization of the sample; not used as an analytic variable.

Item	Participant Response
Current role/title	
Primary clinical unit	
Hospital/site	
Years in pediatric nursing	
Years in current leadership role	
Highest educational degree	
Preferred interview language	Arabic / English (circle one)
Involved in EHR rollout or upgrade?	Yes / No
Involved in telehealth implementation?	Yes / No

Involved in device integration/decision support configuration?	Yes / No
Self-rated digital proficiency (1 = lowest, 5 = highest)	1 – 2 – 3 – 4 – 5 (circle one)

Note on use in analysis. The interview guide was used flexibly, consistent with IPA’s emphasis on following participants’ accounts and attending to emergent meanings. Question order and emphasis varied across participants depending on the direction of each conversation. The guide was not administered as a fixed survey. Interview 2 was further individualized based on the PI's review of each participant’s journal entries prior to the session. Arabic interviews were coded in Arabic; English translations supported team discussion and are used for reported quotations.
