# Supplementary material for: Digital Competencies for Pediatric Nurse Leaders to Sustain Patient- and Family-Centered Care: An Interpretative Phenomenological Analysis
Source: Healthcare (Basel). 2026 May 11;14(10):1303. doi: 10.3390/healthcare14101303 (PMC13205459; doi:10.3390/healthcare14101303)
Supplement: Supplementary file 1 [file healthcare-14-01303-s001.zip › healthcare-4161289-supplementary 2.pdf]

SUPPLEMENTARY MATERIAL S2

Consolidated Criteria for Reporting Qualitative Research (COREQ)

32-Item Checklist — Completed

Digital Competencies for Pediatric Nurse Leaders to Sustain Patient- and Family-Centered Care: An IPA Study

Alaa Hussain Hafiz | Healthcare (MDPI)

Reference. Tong A, Sainsbury P, Craig J. Consolidated criteria for reporting qualitative research (COREQ): a 32-item checklist for interviews and focus groups. Int J Qual Health Care. 2007;19(6):349–357. doi:10.1093/intqhc/mzm042

All 32 COREQ items are addressed below. Responses are grounded in the study methods, procedures, and analytic approach described in the manuscript. Column headers: No. = item number; COREQ Item = standardized item name; Guiding Question = standard prompt from Tong et al. (2007); Response: This Study = specific response for this study.

No.	COREQ Item	Guiding Question	Response: This Study
**Domain 1. Research Team and Reflexivity — Personal Characteristics**			
1	Interviewer/facilitator	Who conducted the interview or focus group?	All interviews were conducted by the principal investigator (PI), Alaa Hussain Hafiz, a pediatric nurse educator and faculty member at King Abdulaziz University.
2	Credentials	What were the researcher's credentials (e.g., PhD, MD)?	The PI holds a nursing doctorate (PhD) and academic faculty appointment in maternity and child health nursing.
3	Occupation	What was the researcher's occupation at the time of the study?	Academic faculty member and pediatric nurse educator, King Abdulaziz University, Jeddah, Saudi Arabia.
4	Gender	Was the researcher male or female?	Not reported. Gender-concordant interviewing was offered to participants upon request to support cultural accommodation.
5	Experience and training	What experience or training did the researcher have?	The PI has expertise in pediatric nursing education and qualitative research. IPA training and prior qualitative research experience informed study design, analysis, and reflexive practice.
**Domain 1 (continued) Relationship with Participants**			
6	Relationship established	Was a relationship established prior to study commencement?	No prior researcher–participant relationship existed. Recruitment proceeded via opt-in: nursing leaders circulated study invitations, and interested individuals contacted the PI directly, preserving voluntariness.
7	Participant's knowledge of the interviewer	What did participants know about the researcher?	Participants were informed of the PI's academic role as a pediatric nurse educator and faculty member embedded in the same

No.	COREQ Item	Guiding Question	Response: This Study
			regional healthcare system, as detailed in the participant information sheet.
8	Interviewer characteristics	What characteristics were reported about the interviewer/facilitator?	The PI's identity as a pediatric nurse educator and faculty member in the regional healthcare system is acknowledged. Potential familiarity effects (facilitating rapport but requiring active reflexive bracketing of normative assumptions about 'correct' digital practice) were addressed in reflexive memos and detailed in Section 2.6 of the manuscript.
**Domain 2. Study Design — Theoretical Framework**			
9	Methodological orientation and theory	What methodological orientation was stated to underpin the study?	Interpretative Phenomenological Analysis (IPA), informed by Heideggerian hermeneutic phenomenology. Recognizes experience as interpreted through professional, cultural, and technological horizons; meaning is co-constructed through engagement with participants' accounts. Theoretical contextualization (Rogers' Diffusion of Innovation; Benner's novice-to-expert) was applied post-idiographically, after themes stabilized.
**Domain 2 (continued) — Participant Selection**			
10	Sampling	How were participants selected?	Purposive maximum-variation sampling across three hospitals selected from a sampling frame of 12 pediatric facilities, using predefined criteria for digital maturity and strata for leadership tier and clinical area.
11	Method of approach	How were participants approached?	Nursing leaders circulated study invitations. Participation was strictly opt-in; interested individuals contacted the PI directly to preserve voluntariness and reduce gatekeeper influence.
12	Sample size	How many participants were in the study?	N = 10 pediatric nurse leaders. Sample sufficiency was determined by attainment of idiographic depth within cases and stabilization of cross-case patterning, consistent with IPA methodology rather than statistical saturation criteria.
13	Non-participation	How many people refused to participate or dropped out?	All recruited participants completed the full protocol (two interviews and four-week reflective journaling). No dropouts occurred. The number who declined initial contact is not available, as recruitment proceeded via opt-in.
**Domain 2 (continued) Setting**			
14	Setting of data collection	Where was the data collected?	Private rooms within each of the three participating hospitals were selected to ensure confidentiality and minimize power dynamics.

No.	COREQ Item	Guiding Question	Response: This Study
			Locations were neutral relative to participants' supervisory relationships.
15	Presence of non-participants	Was anyone else present besides the researcher and participants?	No. Interviews were conducted with the participant and PI only; the private room setting was specifically selected to exclude third parties.
16	Description of sample	What are the important characteristics of the sample?	Participants comprised 8 women and 2 men; age range 28–54 years (median 39); pediatric nursing experience 5–26 years (median 13); leadership experience 2–15 years (median 6). Roles included charge nurses (n=4), unit managers (n=3), clinical nurse specialists (n=2), and one nursing director. Sites: Public Tertiary A (n=4), Public General B (n=3), Private Tertiary C (n=3). See Table 1 of the manuscript.
**Domain 2 (continued) Data Collection**			
17	Interview guide	Were questions, prompts, guides provided by the authors?	Yes. A semi-structured interview guide was used across two in-depth interviews. The complete guide, including Interview 1 and Interview 2 questions, probing questions, and the four-week reflective journaling protocol, is provided as Supplementary Material S1.
18	Repeat interviews	Were repeat interviews carried out? If yes, how many?	Yes. Each participant completed two in-depth interviews (60–90 min each), scheduled approximately four weeks apart (range 3–5 weeks). Total: 20 interviews across the 10 participants.
19	Audio/visual recording	Did the research use audio or visual recording to collect the data?	Yes. All interviews were audio-recorded with participants' written consent. Recordings were accompanied by researcher field notes capturing contextual cues and reflexive observations.
20	Field notes	Were field notes made during and/or after the interview?	Yes. The PI maintained field notes during and immediately after each interview, documenting contextual cues, non-verbal observations, and reflexive responses. These informed IPA initial noting and reflexive memos.
21	Duration	What was the duration of the interviews or focus group?	Individual interviews: 60–90 minutes each. Data collection spanned approximately five months overall (recruitment: two months; data collection, including journaling period: approximately five months).
22	Data saturation	Was data saturation discussed?	Sample sufficiency in IPA is determined by idiographic depth within cases and the stabilization of cross-case patterning, rather than by conventional saturation criteria. Stabilization was assessed after completing idiographic closure for all cases before proceeding to cross-case patterning. This is detailed in Section 2.3 of the manuscript.

No.	COREQ Item	Guiding Question	Response: This Study
23	Transcripts returned	Were transcripts returned to participants for comment and/or correction?	Transcripts were not routinely returned to participants as a member-checking step, consistent with IPA's analytic emphasis on interpretive engagement with data rather than respondent validation. Trustworthiness was instead pursued through prolonged engagement, reflexive memoing, audit trails, independent analytic review, and systematic searching for disconfirming evidence.
**Domain 3. Analysis and Findings — Data Analysis**			
24	Number of data coders	How many data coders coded the data?	Primary coding and analysis were conducted by the PI. A second qualitative researcher with IPA expertise independently reviewed analytic memos and cross-case theme structures, providing an external check on interpretive moves and strengthening collaborative validity.
25	Description of the coding tree	Did the authors provide a description of the coding tree?	Yes. The analytic process is described in Section 2.7 of the manuscript: idiographic noting at descriptive, linguistic, and conceptual levels; within-case emergent theme crafting using IPA clustering techniques (abstraction, subsumption, contextualization, polarization); superordinate/subordinate theme structures; then cross-case patterning. Table 2 presents the master theme table with subthemes and breadth indicators.
26	Derivation of themes	Were themes identified in advance or derived from the data?	Themes were derived inductively from the data, following IPA's idiographic-first procedure. Theoretical lenses (Rogers' Diffusion of Innovation; Benner's novice-to-expert) were engaged only post-idiographically, after themes had stabilized, to situate interpretations within broader scholarship without determining them.
27	Software	What software, if applicable, was used to manage the data?	NVivo 14 was used for data management, coding, and analytic organization. An audit trail comprising analytic memos, decision logs, versioned codebooks, and translation notes was maintained throughout.
28	Participant checking	Did participants provide feedback on the findings?	Formal member checking of findings was not undertaken, consistent with IPA's interpretive epistemological stance. Interpretive accountability was maintained through audit trails, reflexive memoing, independent analytic review, and attention to deviant and disconfirming evidence.
**Domain 3 (continued) — Reporting**			
29	Quotations presented	Were participant quotations presented to	Yes. Illustrative quotations are presented throughout Section 3. Quotations originating in Arabic were translated by a certified

No.	COREQ Item	Guiding Question	Response: This Study
		illustrate the themes/findings?	medical translator, reviewed by a bilingual advisory panel, and lightly edited for readability while retaining meaning. Speaker codes (P01–P10) are used consistently.
30	Data and findings consistent	Was there consistency between the data presented and the findings?	Yes. A clear chain of evidence links raw participant extracts to emergent themes, subthemes, and the interpretive model (Figure 2). Breadth indicators in Table 2 (e.g., “9/10”) are reported descriptively to indicate distribution across cases and do not imply quantitative inference.
31	Clarity of major themes	Were major themes clearly presented in the findings?	Yes. Four superordinate themes, each with related subthemes, are presented in Section 3.3, along with definitions, illustrative quotations, and breadth indicators. Three idiographic vignettes (Section 3.2) precede cross-case synthesis to honor IPA’s idiographic commitment and demonstrate individual meaning-making pathways.
32	Clarity of minor themes	Is there a description of diverse cases or a discussion of minor themes?	Yes. Deviant instances and minority perspectives are discussed in Section 3.3, Section 3.4, and Section 3.6. For example, limits on co-viewing in sensitive situations, variation in comfort with vulnerability disclosure (P09), and the tension between transparency and preparation in information sharing (P01 vs. P07) are explicitly retained and discussed.

Completion note. All 32 COREQ items are addressed. Where full COREQ criteria are not met by standard means (e.g., transcript return, member-checking), the rationale is provided in terms consistent with IPA’s epistemological commitments. The checklist was prepared by the PI and reviewed against the manuscript prior to submission.
